# Exploring the Role of Microplasma for Controlling Cellular Senescence in *Saccharomyces cerevisiae*

**DOI:** 10.3390/molecules30091970

**Published:** 2025-04-29

**Authors:** Farhana Begum, Jaroslav Kristof, Md Jahangir Alam, Abubakar Hamza Sadiq, Mahedi Hasan, Kinoshita Soichiro, Kazuo Shimizu

**Affiliations:** 1 Graduate School of Medical Photonics, Shizuoka University, Hamamatsu 832-8561, Japan; kristof.jaroslav@shizuoka.ac.jp (J.K.); alam.md.jahangir.20@shizuoka.ac.jp (M.J.A.); 2 Graduate School of Science and Technology, Shizuoka University, Hamamatsu 432-8561, Japan; h.s.abubakar.22@shizuoka.ac.jp (A.H.S.); mahedi.hasan.23@shizuoka.ac.jp (M.H.); 3 Graduate School of Integrated Science and Technology, Shizuoka University, Hamamatsu 432-8561, Japan; kinoshita.soichiro.19@shizuoka.ac.jp

**Keywords:** cellular senescence, cold atmospheric microplasma, *saccharomyces cerevisiae*, homeostasis

## Abstract

Cellular senescence plays a pivotal role in aging and stress response mechanisms. Controlling cellular senescence is essential for developing novel techniques to prevent aging or aging-related diseases and promote a healthy lifespan. This study explores the efficiency of cold atmospheric microplasma (CAM) for controlling cellular senescence in yeast *Saccharomyces cerevisiae*. Reactive oxygen and nitrogen species (RONS) generated by CAM influence key processes, such as the regulation of oxidative stress, alterations in membrane potential, and senescence-related epigenetic modifications. As a marker of cellular senescence, the expression of β-galactosidase was assessed in response to different plasma treatments. At a frequency of 1 kHz and a discharge voltage of 5 kV_p-p_, a significant reduction in β-galactosidase activity was observed in cells treated for 10 s and 30 s compared to the control, indicating a reduction in cellular senescence. Additionally, cell viability, metabolic activity, and plasma membrane potential were also found to be higher for the treated cells compared to the control under the same conditions. This study confirms that a physiologically tolerable level of ROS and RNS is sufficient for cellular signaling, but not for damage induction. The findings from this study provide insights on the potential of microplasma as a tool for controlling cellular senescence and the development of therapeutic innovations involving eukaryotic cells.

## 1. Introduction

Senescence is a biological process involving cellular and organismal changes that cause physiological functions to decrease over time, eventually leading to death. Individuals’ physical and mental faculties decrease gradually as a result of accelerated cellular senescence and progressive organ malfunction [1,2]. This degradation is thought to be caused by accumulated damage to molecular and cellular structures, which is also influenced gene expression modification. Senescent cells release a senescence-associated secretory phenotype (SASP), a mixture of inflammatory factors, which can affect neighboring cells and change the microenvironment. In addition to promoting inflammation, the release of the SASP may accelerate the growth of tumors and other age-related diseases [3]. Human somatic cells have a limited ability to divide in culture and eventually reach replicative senescence, a state of permanent proliferation arrest that leads to tissue failure [4]. Biological aging is a key risk factor for the development of human pathologies such as diabetes, cancer, cardiovascular diseases, and neurological diseases [5]. However, controlling cellular senescence can prevent or delay tissue failure and lengthen a person’s healthy lifespan [6]. Senescent cells in mammals exhibit cellular and biochemical changes, including enlarged and flattened morphology, increased ROS production, senescence-associated β-galactosidase activity, and production of the senescence-associated secretory phenotype [7]. In order to study cellular senescence, the yeast *Saccharomyces cerevisiae* has been chosen. *S. cerevisiae* is also known as budding yeast. It is a well-known model organism for studying cellular senescence [8]. Yeast has played a vital role in revealing the molecular genetics underlying numerous fundamental biological processes, including cell cycle regulation [9], protein folding [10,11], intracellular trafficking [12,13], and many more. This small unicellular organism possesses several unique properties, including a rapid generation time and ease of use, as well as benefiting from being able to be analyzed using inexpensive lab equipment and the option to apply powerful genetic approaches, compared to other eukaryotic cells [14]. Furthermore, it is a good model for studying human diseases because it has similarities (homologues and orthologues) with mammalian (including human) cells [15]. Yeast cells age as they divide, just like cultured mammalian cells: the cell size increases, the shape of the cell changes, the cell cycle slows down, and they become sterile [16]. Furthermore, in aged yeast cells, the nucleolus tends to be larger or more fragmented, and the mitochondria become dysfunctional [16,17]. As cells age, their ribosome activity and protein synthesis rate decrease linearly [18]. Oxidative stress, protein aggregation, and extrachromosomal rDNA circles accumulate in mother cells during the budding process, resulting in senescence [16]. However, controlling cellular senescence can mitigate tissue dysfunction and promote a healthy lifespan. In this study, the role of microplasma has been taken into consideration. Microplasma is a type of cold atmospheric plasma that occurs on a microscale. It is a rich source of active species and elements, such as electrons, excited atoms and molecules, ions, reactive oxygen species (ROS), reactive nitrogen species (RNS), and electromagnetic radiation [19,20,21]. The energetic particles and radiation can cause coupling interactions with organisms via particle bombardment and photochemical reactions, resulting in certain beneficial biological effects, such as increased biological activity and stress tolerance [22,23]. Applications of cold atmospheric plasma have gained interest recently because of their numerous potential benefits, including the presence of extremely reactive species like ROS and RNS [24] and because there is no need for expensive vacuum enclosures. Especially in the medical and biological fields, several recent studies have shown that plasma is highly efficient for skin treatment [25,26], transdermal drug delivery [27,28], skin disinfection, treatment of acne or wrinkles [29,30,31,32], and rejuvenation therapy [33]. It is also useful for blood coagulation, wound healing [34], and cancer treatment [35,36]. However, the role of microplasma in controlling cellular senescence remains underexplored. This study focuses on exploring the effect of microplasma for controlling cellular senescence in *Saccharomyces cerevisiae*. In this study, we investigate the efficiency of microplasma in optimum plasma conditions by observing the morphological changes of cells, including cell viability, metabolic activity, and cell membrane potential etc. Furthermore, we seek to understand how microplasma-induced changes can influence yeast cell aging, potentially paving the way for innovative strategies to mitigate senescence and promote cellular rejuvenation.

## 2. Results

### 2.1. Assessing the Optimum Plasma Conditions

**Figure 1:**
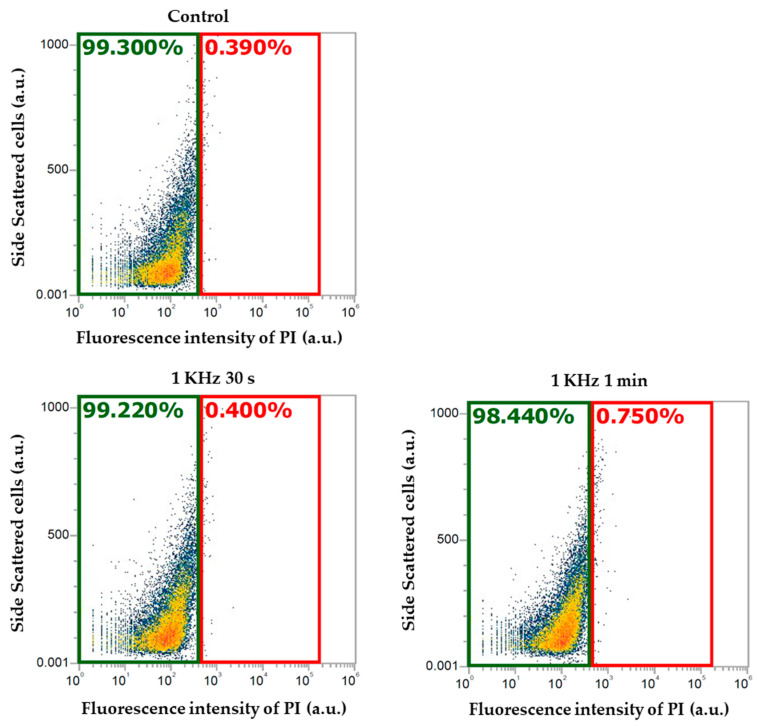

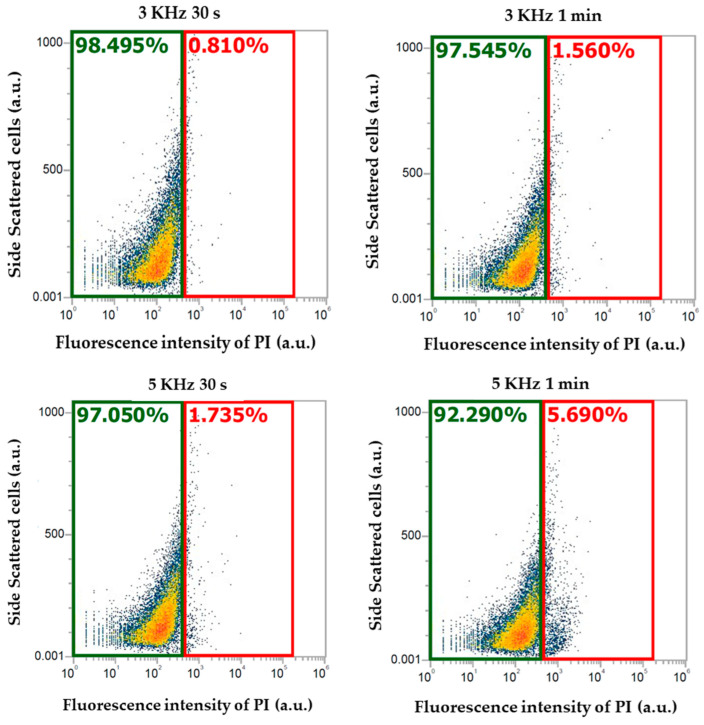
Fluorescence intensity of the cells stained with propidium iodide (PI) observed using flow cytometer. The green area represents the percentage of viable cells, and the red area represents the percentage of dead cells.

**Figure 2:**
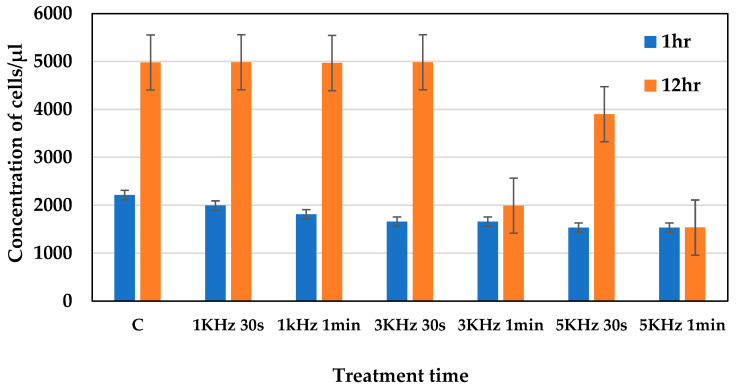
Concentration of cells observed after 1 h and 12 h of plasma treatment.

The optimum plasma conditions were assessed by analyzing the viability of the cells at different frequencies, such as 1 kHz, 3 kHz, and 5 kHz, for treatment times of 30 s and 1 min. The voltage was 5 kV_p-p_ for all the conditions. The fluorescence intensity of the cells, stained with propidium iodide, was observed using flow cytometer (blue laser), at an excitation wavelength of 488 nm and within an emission range of 570–610 nm. Using flow cytometric analysis, we observed that the percentage of dead cells increased as the frequency increased (Figure 1). Furthermore, after 12 h of incubation, the concentration of cells decreased in plasma-treated cells after 1 min of being subject to frequencies of both 3 kHz and 5 kHz plasma conditions. Whereas, the plasma-treated cells subject to a frequency of 1 kHz showed no significant changes in cell concentration compared to the control (Figure 2), which indicates that this condition is optimum for cells, causing no significant stress or damage to the cells.

### 2.2. Senescence-Associated β-Galactosidase Activity

**Figure 3:**
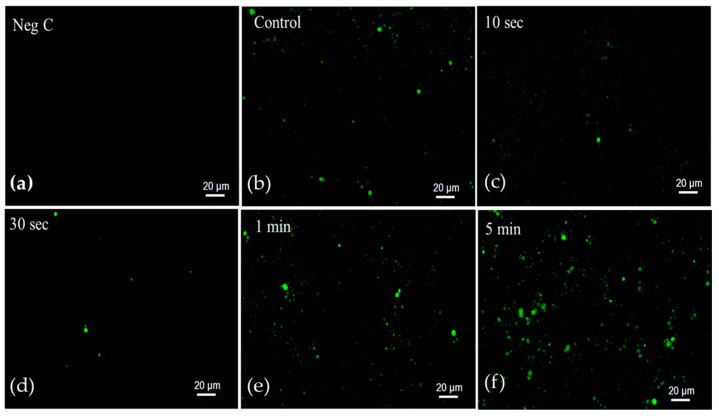
Senescence-associated β-galactosidase activity of *S. cerevisiae* cells observed using a senescence green flow cytometry assay kit with different treatment times. Green fluorescence represents the senescent cells. The images were captured at 20× magnification. The images are as follows: (**a**) non-stained cells, (**b**) untreated stained cells, and (**c**) cells treated with plasma for 10 s, (**d**) 30 s, (**e**) 1 min, and (**f**) 5 min.

As a marker of senescent cells, the β-galactosidase activity of the cells was observed using a senescence green flow cytometry assay kit. At a frequency of 1 kHz and a discharge voltage of 5 kV_p-p_, with different treatment times of 10 s, 30 s, 1 min, and 5 min, plasma was applied to the cells. The fluorescence intensity of the dye was observed under a fluorescence microscope (GFP filter), within an excitation range of 450–490 nm and an emission range of 500–550 nm. As shown in Figure 3, the β-galactosidase activity was observed to be lower in cells treated with plasma for 10 s and 30 s compared to the control, indicating reduced cellular senescence. Whereas, the 1 min and 5 min plasma-treated samples showed a gradual increase in β-galactosidase activity, which indicates a higher level of cellular senescence. 

### 2.3. Measurement of the Cell Size

**Figure 4:**
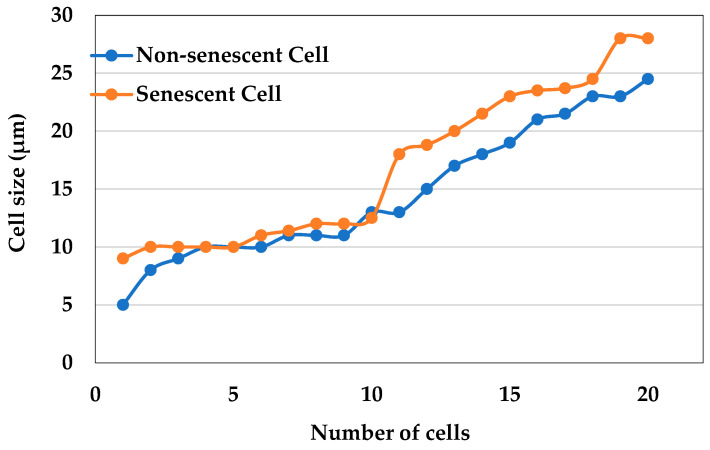
Size of cells in two different cultures, containing non-senescent and senescent cells.

The cell size was measured using two different cultures containing non-senescent and senescent cells and compared with the plasma-treated sample. Three types of cells were analyzed, namely cells without any buds, small-budded cells, and large-budded cells. As shown in Figure 4, the size of non-senescent cells was found to be smaller when compared to the senescent cells. Specially, the size of small-budded and large-budded cells was found to be bigger in the culture containing senescent cells. In the cells treated with plasma for 10 s and 30 s, most of the cell sizes were found to be within the range of non-senescent cells. In the sample treated with plasma for 1 min, the number of bigger cells was moderate. But in sample treated with plasma for 5 min, a large number of the cells were found to be bigger in size and were found to be the same as senescent cells.

### 2.4. Cell Viability Measurement

**Figure 5:**
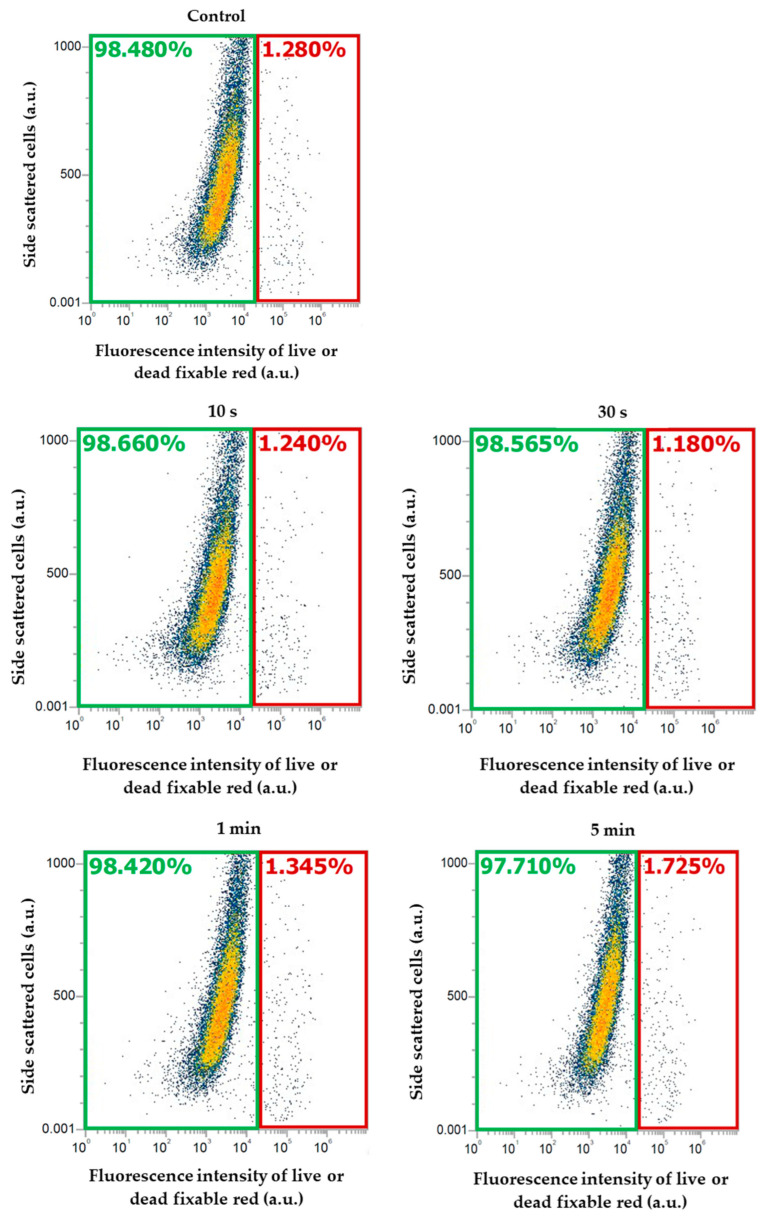
Fluorescence intensity of the cells stained with live or dead fixable red dead cell stain kit, observed using flow cytometry. The green area represents the percentage of viable cells and the red area represents the percentage of dead cells.

After plasma irradiation, the cell viability was analyzed using a live or dead fixable red dead cell stain kit. The fluorescence intensity of the cells stained with this dye was observed using flow cytometer (yellow laser), at an excitation wavelength of 561 nm and within an emission range of 605–628 nm. Here, the x-axis represents the intensity of the cells, and the y-axis SSC (side-scattered cells) represents the complexity of the cells. Within this area, every point represents one cell. Cell viability was found to be higher in all the samples subject to the treatment conditions. But, according to the increase in the treatment time, a slight increase in the percentage of dead cells was observed (Figure 5). 

### 2.5. Measurement of the Metabolic Activity of the Cells

**Figure 6:**
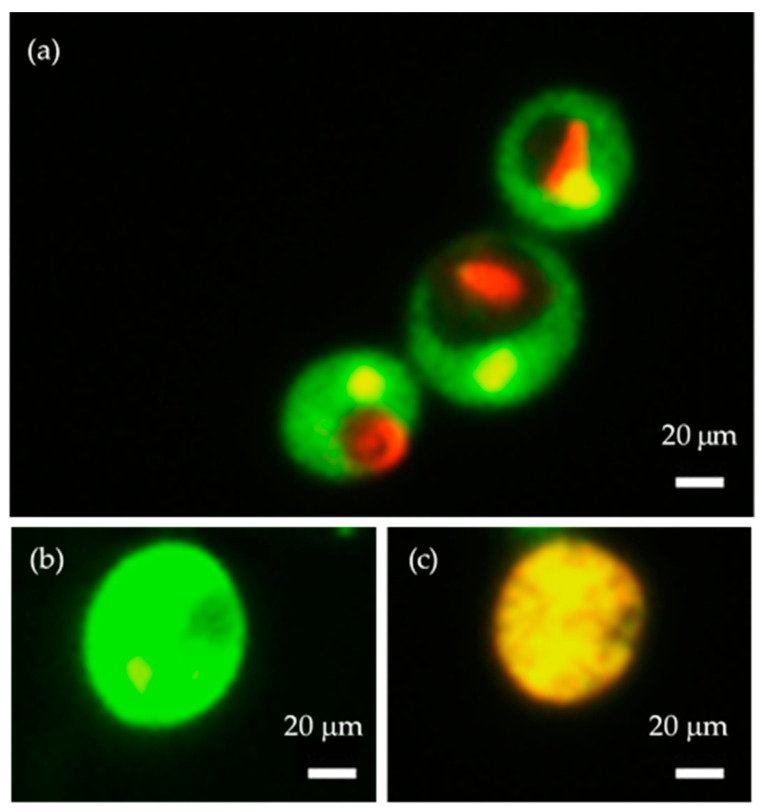
Cells stained with FUN-1 cell stain dye, observed under a fluorescence microscope. Here, the images are as follows: (**a**) intensity of metabolically active cells with a red cylindrical intravacuolar structure (30 s), (**b**) intensity of cells that are alive but not metabolically active (1 min), and (**c**) intensity of dead and metabolically inactive cells (5 min). Images were captured using 100× magnification.

**Figure 7:**
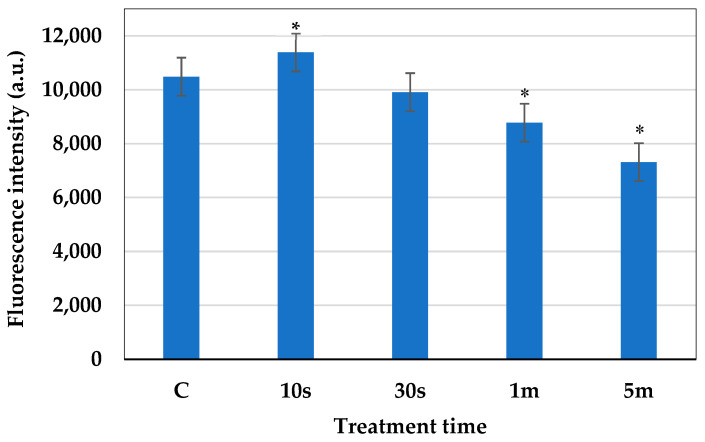
Red fluorescence intensity of the cells after 10 s, 30 s, 1 min, and 5 min of plasma treatment. The statistical differences were considered significant when *p* ≤ 0.05 (*).

The metabolic activity of the plasma-treated cells was analyzed using the FUN-1 dye. After plasma treatment (the plasma condition mentioned in Section 2.1), the red fluorescence intensity of metabolically active cells was observed using a microplate reader, at an excitation wavelength of 520 nm and an emission wavelength of 620 nm. The structure of metabolically active cells was observed under a fluorescence microscope. The images were captured for both green (GFP filter, the excitation range was 450–490 nm and the emission range was 500–550 nm) and red fluorescence (TRICT filter, the excitation range was 520–570 nm and the emission range was 570–640 nm). The overlay image based on both green and red fluorescence shows that the cells that are metabolically active formed a cylindrical intra-vacuolar structure that emits red fluorescence (Figure 6). The intensity of the red fluorescence of the cells is shown in Figure 7. In the cells treated with plasma for 10 s, the intensity of the red fluorescence was found to be higher than the other samples, which indicates the presence of a higher amount of metabolically active cells. In the cells treated with plasma for 30 s, the amount of metabolically active cells was decreased a little, but was not statistically significant. In the samples subjected to 1 min and 5 min of treatment, the number of metabolically active cells was decreased significantly compared to the control.

### 2.6. Measurement of the Cell Membrane Potential

**Figure 8:**
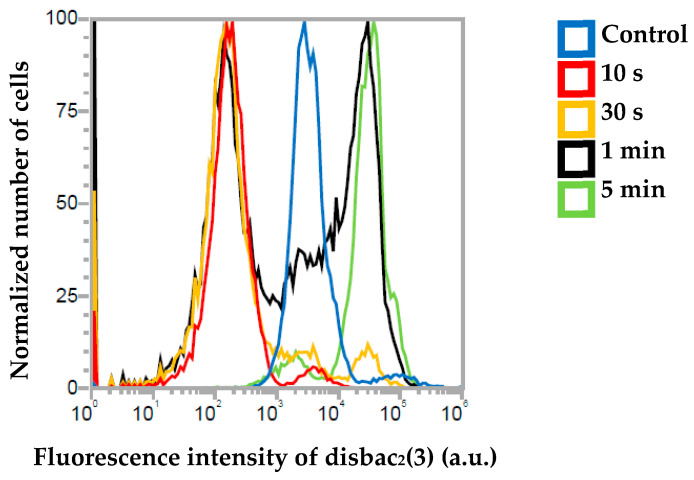
Fluorescence intensity of non-treated cells and cells treated with plasma for 10 s, 30 s, 1 min, and 5 min, observed using a flow cytometer.

The cell membrane potential represents the cell membrane integrity of cells. In normal viable cells, the cell membrane maintains stable integrity to regulate the cellular function [37]. The measurement of the cell membrane potential enabled us to assess the effect of plasma irradiation on cell membrane integrity. The resting membrane potential is a state of electrical potential difference across the plasma membrane, when it is in a non-excited state. An increase in fluorescence intensity represents an increase in the cell membrane potential and a decrease in fluorescence intensity represents a decrease in the cell membrane potential. An increase in the positivity of the cell membrane potential refers to its depolarization, whereas hyperpolarization refers to an increase in the negativity of the cell membrane potential [38]. Figure 8 shows the changes in the membrane potential of the cells in response to plasma treatment. Wherein the control sample shows no changes in its fluorescence intensity, which indicates that the cells are in a resting potential state. The cells treated with plasma for 10 s and 30 s show a decrease in fluorescence intensity that indicates hyperpolarization. Whereas, in the cells treated with plasma for 1 min, some cells are in a depolarization state and some are in a hyperpolarization state. However, in the cells treated with plasma for 5 min, an increase in fluorescence intensity was shown, which indicates that the cells are in a depolarization state.

### 2.7. Amount of Intracellular ROS

**Figure 9:**
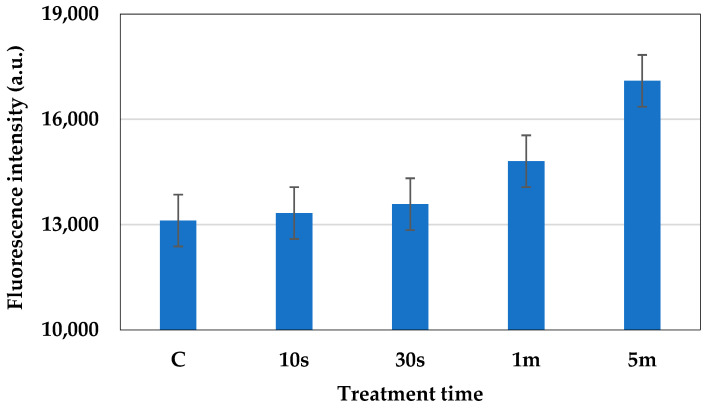
Fluorescence intensity of intracellular ROS in the cells treated with plasma for 10 s, 30 s, 1 min, and 5 min.

The amount of intracellular reactive oxygen species was measured via a microplate reader, using a ROS assay kit-highly sensitive DCFH-DA dye, at an excitation wavelength of 485 nm and an emission wavelength of 535 nm. After plasma irradiation on cells, it was observed that the amount of intracellular ROS was significantly increased in the cells treated with plasma for 1 min and 5 min (Figure 9).

### 2.8. Concentration of Active Nitrogen Species

**Figure 10:**
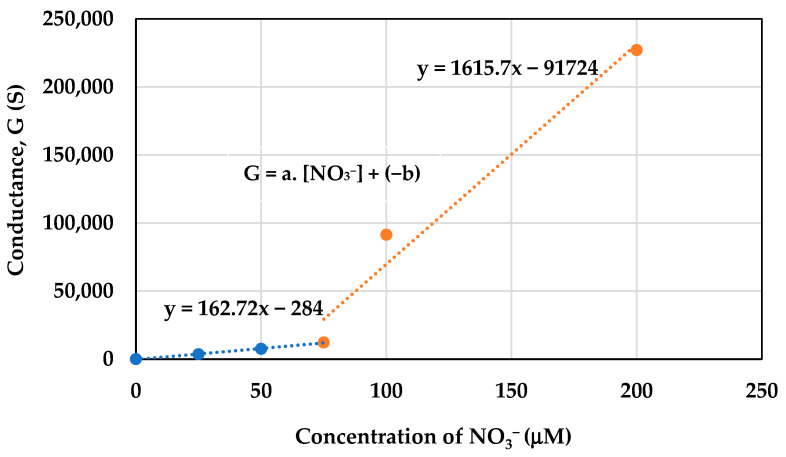
Calibration curve of standard KNO_3_ solution.

The concentration of active nitrogen species generated at the time of plasma irradiation was observed using ion chromatography. The concentration of NO_3_^−^ was determined based on the calibration curve of a standard KNO_3_ solution (Figure 10). Wherein the x-axis represents the concentration of NO_3_^−^ and the y-axis represents conductance. The curve is generated by plotting known standard concentrations of KNO_3_ against their corresponding conductance. From the calibration curve, we noted two linear lines in response to different concentration of NO_3_^−^ solution that best fitted to the data points. The equation for this line is y = mx + c; according to which, G = a. [NO_3_] + (−b), is used to calculate the unknown concentration of the generated NO_3_^−^ ion in the plasma-treated sample.

**Figure 11:**
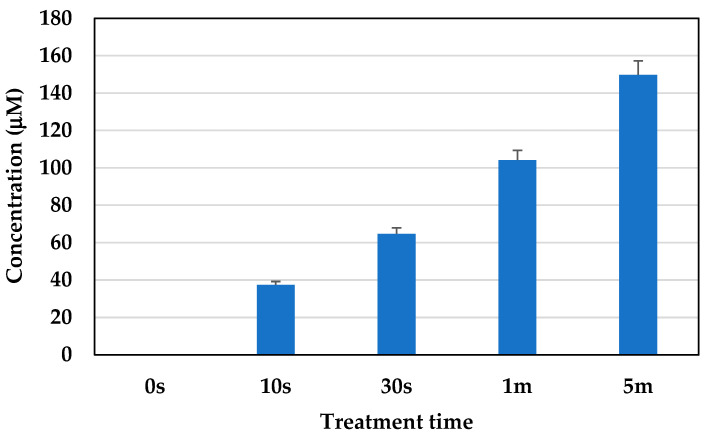
Concentration of NO_3_^−^ generated in the samples under the treatment conditions.

As shown in Figure 11, the production of NO_3_^−^ was observed for different treatment times. Wherein the NO_3_^−^ concentration was found to be 37 μM, 64 μM, 104 μM, and 149 μM for the cells treated with plasma for 10 s, 30 s, 1 min, and 5 min, respectively.

## 3. Discussion

This study investigated the effect of cold atmospheric microplasma in regard to controlling cellular senescence, using *S. cerevisiae* yeast cells. The results of this investigation showed that cold microplasma has a very promising potential effect in regard to controlling cellular senescence, while maintaining the viability of the cells. The observations carried out in this study of cellular senescence showed an increase in the activity of β-galactosides in senescent cells and a decrease in such activity in non-senescent cells.

According to Gary et al. 2005 [39], an increased amount of enzymatic activity was observed in cells when they became senescent. Under acidic pH conditions, the hydrolase enzyme resides in lysosomes and converts β-galactosides into monosaccharides. The β-gal activity was found to be twice as high in senescent cells compared to pre-senescent cells. An increase in the cell size was observed in senescent cells that also supports the previous findings in [40]. In the cells treated with plasma for 10 s and 30 s, more metabolically active cells were identified compared to the cells treated with plasma for 1 min and 5 min. The metabolic activity of the cell indicates its capability for normal cellular functioning [41]. It also has a relationship with cellular senescence, as senescent cells are not completely metabolically inactive, but exhibit a significant decrease in their overall metabolic activity compared to healthy, proliferating cells. This state is characterized by impaired mitochondrial function, altered utilization of metabolic substrates, reduced ATP production, and contributes to the accumulation of cellular damage and the release of pro-inflammatory factors associated with aging [42,43]. The higher cell viability under plasma treatment conditions indicate its potential for controlling cellular senescence without causing any harm to the cells. The plasma membrane potential supports the findings on the viability and metabolic activity of the cells, as only live cells are able to maintain membrane potential. However, membrane depolarization indicates a decrease in cell activity, but it does not indicate cell death [44]. The discharge of microplasma in air generates ROS (O, O_2_, and O_3_) and RNS (NO, NO_2_, and ONOO-). The presence of water vapor aids in the discharge of microplasma in room-temperature air, resulting in the generation of H_2_O_2_, OH, HNO_3_, and HNO_2_. The electron-impact dissociation of O_2_, N_2_, and H_2_O (humidity of room-temperature air) initiates the mechanisms that cause microplasma to generate ROS and RNS. At this point, some ROS and RNS are produced, along with the reactive products that are also required to create further ROS and RNS [45]. The excited states of O_2_, N_2_, and H_2_O are caused by inelastic collisions, which are the primary source of electron energy loss. NO is formed from the excited electrical state of N_2_ (N_2_^∗^), which is dissociatively quenched by O_2_ to yield O atoms and also takes part in reactions with O. The three-body reaction of O_2_ and O produces ozone (O_3_) [46,47]. ROS, RON, and also UV light affect the cell envelope and the internal components of cells. The intricate process according to which a living cell interacts with plasma is influenced by a number of factors, including the dose of plasma, the size and type of cell, biochemical variations in the cell membrane, metabolic rates, and variations in the cell cycle. Cells with relatively high metabolic rates can selectively interact with intermediate amounts of plasma, which effectively controls cell death without necrosis [48,49]. The generation of O_3_ during the plasma treatment was measured using an ozone monitor, connected to the microplasma set up. The concentration of ozone generated during treatment was 1.9 ppm, 33.2 ppm, 56.5 ppm, and 154.3 ppm for the cells treated with plasma for 10 s, 30 s, 1 min, and 5 min, respectively. The amount of NO_3_^−^ was measured as 37 μM, 64 μM, 104 μM, and 149 μM for the samples treated with plasma for 10 s, 30 s, 1 min, and 5 min, respectively.

A minimal amount of intracellular ROS and active nitrogen species was detected during samples subject to 10 s and 30 s of plasma treatment. The findings from previous studies showed that a higher amount of ROS/RNS triggered the accumulation of oxidative damage in biomolecules, which causes premature senescence [50,51]. But, in regard to the relevant physiological levels, ROS/RNS contribute through the provision of important proliferative cascades and function as signaling molecules [52,53,54]. RNS, along with ROS, have been found to act as secondary messengers to help maintain homeostasis at the cellular level [55]. This emphasizes the importance of the findings in the present study, namely that the optimum amounts of ROS and RNS play a significant role in controlling cellular senescence. By maintaining the optimum plasma conditions, including the frequency, voltage, and time of exposure, cellular senescence can be controlled without causing any harm to normal cells.

## 4. Materials and Methods

### 4.1. Materials Used

*Saccharomyces cerevisiae* cell Strain BY4741 (*MATa leu2Δ0 ura3Δ0 his3-Δ1 met15Δ0*) was purchased from the national bio-resource project (NBRP), Kyoto, Japan. BD Difco™ YPD (Yeast Peptone Dextrose) Broth was purchased from Becton, Dickinson and Company, Franklin Lakes, NJ, USA. Phosphate buffered saline (PBS) was purchased from Shimadzu Diagnostics Corporation (Tokyo, Japan). An orbital shaker (model OS- 20 pro) was purchased from JOANLAB, Huzhou, China. The microplate reader, Spectra Fluor Plus, was purchased from Tecan (Männedorf, Switzerland). A high-performance ion chromatograph (model LC-20 AD) was purchased from Shimadzu Corporation (Tokyo, Japan). An Attune NxT Flow Cytometer was purchased from Thermo Fisher Scientific, Waltham, MA, USA. A Keyence Florescence Microscope All-in-one BZ-x800 was purchased from Keyence Corporation, Osaka, Japan. BD Pharmingen™ Propidium Iodide staining solution was purchased from BD Biosciences, San Jose, CA, USA. A LIVE/DEAD™ fixable red dead cell stain kit, FUN™-1 Cell Stain, and DiSBAC_2_(3) (Bis-(1,3-Diethylthiobarbituric Acid) Trimethine Oxonol) was purchased from Thermo Fisher Scientific, Waltham, MA, USA. Dojindo’s ROS Assay Kit, Highly Sensitive DCFH-DA, was purchased from Dojindo laboratories, Tokyo, Japan.

### 4.2. Cell Culture Conditions

*S. cerevisiae* cells were cultured in a yeast extract peptone dextrose (YPD) medium, with a composition of 10 g of yeast extract, 20 g of peptone, and 20 g of dextrose. The cells were cultured in an incubator at 30 °C, with continuous shaking at 200 rpm.

### 4.3. DBD Microplasma Set Up

**Figure 12:**
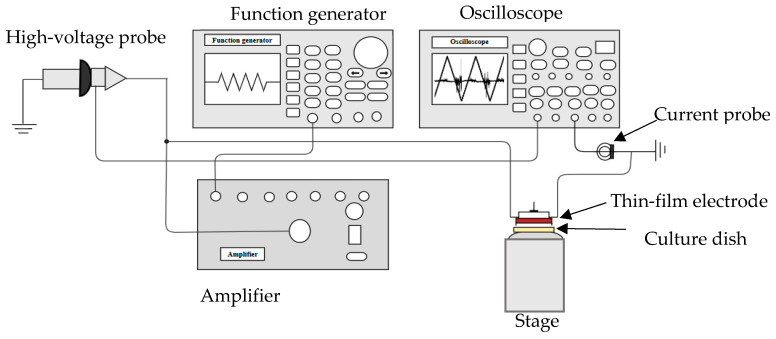
Schematic diagram of the experimental set up for microplasma treatment.

The dielectric barrier discharge (DBD) microplasma set up used for this study is shown in Figure 12. A thin-film electrode was used to generate DBD plasma under atmospheric conditions [47]. At the time of irradiation, pure atmospheric gas was used at a flow rate of 0.2 L/min. The plasma discharge was generated (the plasma condition mentioned in Section 4.4) using a function generator (AFG3102, Tektronix, Beaverton, OR, USA) and a triangle (positive and negative) waveform was set, which was subsequently amplified by a high-voltage amplifier (5/80, Trek, New York, NY, USA). A high-voltage probe (model P6105A, Tektronix), connected to an oscilloscope (model TDS2014B, Tektronix), was used to measure the discharge voltage. A Pearson current monitor was also connected to the oscilloscope, to measure the discharge current.

### 4.4. Microplasma Electrode

**Figure 13:**
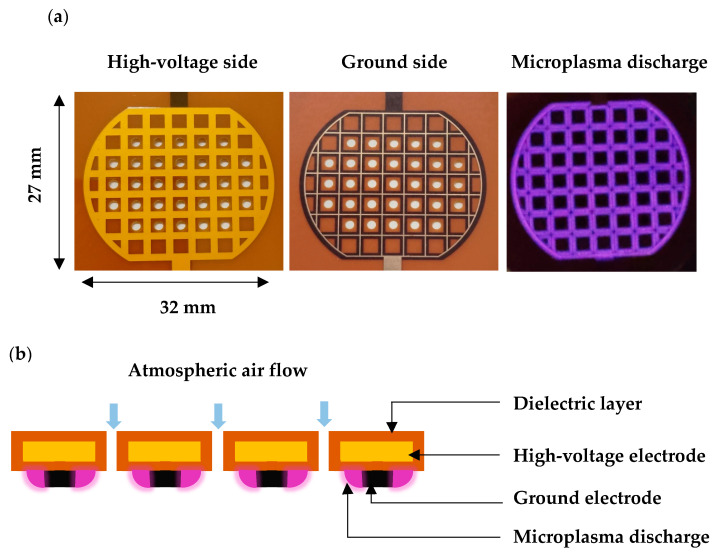
Microplasma electrode: (**a**) images showing the back view (high-voltage side), front view (ground side), and electrode during discharge; and (**b**) cross-section view of microplasma electrode.

As shown in Figure 13, this experiment used a surface DBD-type electrode, which is made up of a grid-shaped wire that is 1.2 mm wide on the high-voltage side and 0.2 mm wide on the ground side. The wires are separated by a 25 μm thick dielectric substance. The film, made of polyamide, is employed as a dielectric. The high-voltage electrode is surrounded by a 50 μm thick dielectric layer. The electrodes are 30 mm in diameter, and the grid pattern is arranged with a 3.5 mm space between them. The electrode comprises 31 perforated holes, each around 2 mm in diameter, to allow the gas to flow and to transport the active species generated by the microplasma to the desired location. Figure 13a illustrates the microplasma discharge that developed close to the ground electrode’s grid.

### 4.5. Plasma Conditions and Treatment

**Figure 14:**
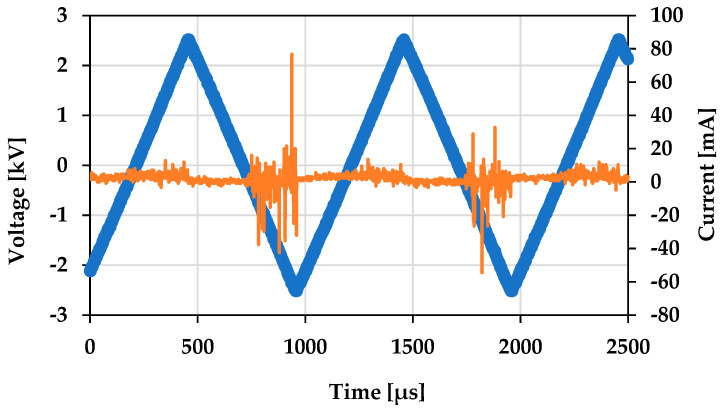
Voltage and current of microplasma discharge waveform. Voltage is showing in blue and current is showing in orange.

**Figure 15:**
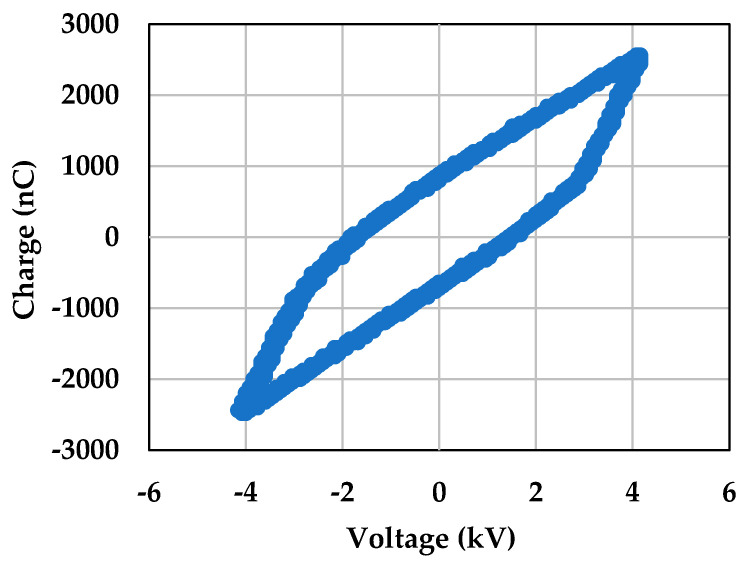
Lissajous figure representing the Q-V plot of the microplasma treatment at 5 kV_p-p_ voltage and 1 kHz frequency.

For irradiating plasma onto *S. cerevisiae* cells, the cells were transferred to Petri dishes (60 mm × 15 mm), with the concentration of the medium being 1 × 10^6^ cells/mL. The concentration of the cells was measured using flow cytometry. A direct plasma treatment method was used for treating the cells with microplasma. When samples undergo direct plasma treatment, they are usually exposed to the complete spectrum of radiation generated in active plasma regions, as well as electric fields, energetic electrons and ions, excited species, and radicals [56]. A discharge voltage frequency of 1 kHz and 5 kV was used for the treatment. The distance between the surface of the cell medium and the thin-film electrode was approximately 3 mm. The cells were treated with microplasma for a duration of 10 s, 30 s, 1 min, and 5 min. The same conditions were used for all the experiments. The discharge voltage and current characteristics of microplasma are shown in Figure 14. The Lissajous figure of the DBD source with this particular electrode configuration is shown in Figure 15, representing the relationship between the instantaneous charge (Q) and voltage (V) of the DBD plasma in the above treatment conditions.

### 4.6. Measurement of the Cellular Senescence

Cellular senescence causes different morphological and physiological changes in cells. In this study, in response to the microplasma treatment, cellular senescence was analyzed by observing the changes in the senescence-associated β-galactosidase activity, changes in the cell size, and metabolic activity of the cells.

#### 4.6.1. Measurement of SA β-Galactosidase Activity

A CellEvent™ senescence green flow cytometry assay kit (Thermo Fisher Scientific, Waltham, MA, USA) was used for the detection of senescent cells. This is a fluorescent-based reagent that contains two galactoside moieties that work by specifically binding to β-galactosidase. The enzyme-cleaved product is retained in the cell due to covalent binding of intracellular proteins and emits a fluorescence signal that has an excitation/emission maxima of 490/514 nm. This kit contains a CellEvent™ senescence green probe (1000×) and CellEvent™ senescence buffer. A working solution was prepared at the time of staining and was diluted (1:500). The senescence green probe was placed into the senescence buffer. The cells were subjected to plasma treatment for the time duration stated previously (Section 4.4) and incubated for 4 h to grow and divide in the culture medium. After incubation, the cells were washed with PBS and resuspended in PBS to a concentration of about 1 × 10^6^ cells per 100 µL. A 100 µL aliquot of the cell suspension was placed into the flow tubes. The tubes were centrifuged and then the media were discarded. The cells were resuspended in 2% paraformaldehyde (with 100 µL of fixation solution) and incubated for 15 min at room temperature, protected from light. The cells were washed with PBS to remove the fixation solution. Then, the cells were resuspended in 100 µL of working solution that contained the dye. The cells were incubated for 2 h at 30 °C without CO_2_, and protected from light. After incubation, the working solution was removed and, again, the cells were washed with PBS. The cells were resuspended in PBS and then analyzed under a fluorescence microscope (Keyence All-in-one BZ-X800). For the microscopic observations, a GFP (green fluorescent protein) filter was used that has an excitation range of 450–490 nm and an emission range of 500–550 nm. Non-irradiated cells stained with the dye were used as controls. Triplicate experiments were conducted for each sample to ensure reproducibility.

#### 4.6.2. Cell Size

A phase contrast image of freshly cultured non-senescent cells and old cultured senescent cells was taken under microscope, and the cell size was measured using ImageJ software (ImageJ version 1.54g, Wayne Rasband and Contributors National Institute of Health, Bethesda, MD, USA). Cells from different stages (unbudded, small-budded and large-budded cells) were chosen for measurement. The comparative cell size was then analyzed using plasma-irradiated cells.

### 4.7. Cell Viability

Two types of dye were used to observe the viability of the cells in different treatment conditions. Propidium iodide (PI) dye was used to observe the viability of the cells treated with different frequencies and a flow cytometer was used. The fluorescent dye, PI, can attach to DNA, but it cannot penetrate cells with intact plasma membranes. When the membrane is broken, this feature allows PI to distinguish between dead cells and living cells with undamaged membranes. PI can be used to measure cell death in flow cytometric analysis, since it is excited by wavelengths between 400 and 600 nm and emits wavelengths of between 600 and 700 nm [57,58]. Again, cell viability was measured with a LIVE/DEAD™ fixable red dead cell stain kit for the specific treatment condition used in this study, using an Attune NxT Flow Cytometer (Thermo Fisher Scientific, Tokyo, Japan). In cells that have a compromised membrane, the dye reacts with free amines both in the cell interior and on the cell surface and yields intense fluorescent staining. In viable cells, the dye’s reactivity is restricted to cell-surface amines, resulting in less intense fluorescence. The difference in intensity is typically greater than 50-fold between live and dead cells, allowing for easy discrimination. This dye is excited by wavelengths of 488–595 nm and an emission of ~615 nm. After plasma treatment, the cells were incubated for 30 min. Moreover, 1 mL of the cell suspension sample containing 1 × 10^6^ cells was centrifuged. The supernatant was discarded and the cells were washed once with 1 mL of PBS. The cells were resuspended in 1 mL of PBS. Then, 1 µL of the fluorescent reactive LIVE/DEAD™ fixable red dye was added to 1 mL of the cell suspension and mixed well. In the case of PI, 5 µL of dye was added to the cell suspension. The cells were incubated at room temperature for 30 min, while being protected from light. After that, the cell viability was measured using a blue laser in terms of flow cytometry, at an excitation wavelength of 488 nm and an emission range of 570–610 nm. Non-irradiated cells stained with this dye were used as controls. Triplicate experiments for each sample were conducted to ensure reproducibility.

### 4.8. Metabolic Activity of the Cells

The metabolic activity of the cells was measured with FUN™-1 cell stain dye. This dye has the ability to passively diffuse into a wide variety of cell types. It initially stains the cytoplasm with a diffusely distributed green fluorescence. In live cells, the dye leads to the formation of distinct vacuolar structures with compact morphology that exhibit significant red fluorescence, accompanied by a decrease in green cytoplasmic fluorescence. The formation of intravacuolar structures requires both cell membrane integrity and metabolic competence. Whereas dead cells emit a bright yellow–green fluorescence, with no discernible red structures [59]. For measuring the metabolic activity of plasma-irradiated yeast cells, a suspension of 1 × 10^6^ cell/mL was stained with 1 µL of FUN-1 cell stain dye (the final concentration was 5 μM). The solution was mixed thoroughly and incubated at 30 °C in the dark for 30 min. Non-irradiated cells stained with the dye were used as controls. The fluorescence intensity was measured using a microplate reader for both green (excitation/emission wavelength of 485 nm/535 nm) and red (excitation/emission wavelength of 520 nm/620 nm) fluorescence. The structure of metabolically active, inactive, and dead cells was also observed under a fluorescence microscope. A fluorescence microscope was used to observe both green fluorescence (using a GFP filter, excitation range of 450–490 nm and emission range of 500–550 nm) and red fluorescence (TRICT filter, excitation range of 520–570 nm and emission range of 570–640 nm).

### 4.9. Cell Membrane Potential

Cell membrane potential was analyzed via flow cytometry, using DiSBAC_2_(3) dye, at an excitation wavelength of 561 nm and within an emission range of 577–593 nm. DiSBAC_2_(3) dye is a negatively charged dye used to analyze the cell membrane potential or charge of a membrane. In normal cells, the inside of the cell is negatively charged. When the membrane potential decreases, it becomes less negative inside of the cell, and the membrane becomes depolarized. This dye can enter depolarized cells, wherein it binds to intracellular proteins or membranes and exhibits enhanced fluorescence. Increased depolarization results in an additional influx of the anionic dye and an increase in fluorescence. Conversely, hyperpolarization is indicated by a decrease in fluorescence [60].

### 4.10. Measurement of Intracellular ROS

The reactive oxygen species (ROS) was measured using the ROS Assay Kit (Dojindo, Kumamoto, Japan), highly sensitive DCFH-DA. When DCFH-DA is taken into the cell, it is deacetylated by esterase and transformed into DCFH. When the DCFH is promptly oxidized by ROS, it changes to a fluorescent DCF. The measured wavelength was at excitation of 485 nm and an emission of 535 nm. The kit contains highly sensitive DCFH-DA dye and loading buffer (10×). The highly sensitive DCFH-DA dye was diluted (1:1000) in a reconstituted loading buffer solution to prepare the working solution. The 10× loading buffer (1:10) was diluted with double-deionized water. The *S. cerevisiae* cells were cultured and incubated at 30 °C. After culturing for 24 h, the culture was washed two times with PBS and the medium was changed. Then, the dye was added to the culture plate and plasma irradiation was performed with the DBD microplasma. After 10 min of incubation, the culture plate was washed twice with PBS and the fluorescence intensity was measured using a microplate reader. Triplicate experiments were conducted for each sample.

### 4.11. Measurement of Active Nitrogen Species

The active nitrogen species generated at the time of microplasma treatment were measured using ion chromatography (model shin-pack IC- SA3, Shimadzu Corporation, Kyoto, Japan; column length 250 mm, diameter 4 mm, flow rate of mobile phase was 0.8 mL/min, column temperature was 45 °C, composition of column was 3.6 mmol/L of NaHCO_3_ in distilled water). The plasma was energized in distilled water. The concentration of NO_3_^−^ was calculated based on a standard solution of potassium nitrate (KNO_3_). Then, the concentration of NO_3_^−^ generated by the plasma-treated sample was determined by comparing the results with the calibration curve.

## 5. Conclusions

In this study, we investigated the role of cold atmospheric microplasma in regard to controlling cellular senescence in yeast cells. For this purpose, the important hallmark of cellular senescence, senescence-associated β-galactosidase activity, was assessed under different plasma conditions. With a 1 kHz frequency and a 5 kV discharge voltage, a treatment time of 10 s and 30 s, and by using cold atmospheric microplasma, a significant decrease in cellular senescence was observed in yeast cells, while the cell viability and metabolic activity of the cells was well-maintained. The cell membrane integrity was also found to be higher in the samples subject to this treatment conditions compared to the control. The detection of a minimal amount of ROS and active nitrogen species in the samples subject to the treatment conditions confirms its role in controlling cellular senescence. In a future study, we will also analyze the effect of microplasma on mammalian cells. Following this, a clinical trial will also be conducted on a mouse model to evaluate its efficiency for practical applications. The results of this study will pave the way for the development of a new therapeutic technique for treating senescence-related diseases.

## Data Availability

The original contributions presented in this study are included in the article. Further inquiries can be directed to the corresponding author(s).
